# Exploring the potential of *Gonolobus condurango* as a histone deacetylase inhibitor in triple-negative breast cancer cell lines: in vitro study

**DOI:** 10.1186/s12906-025-04896-w

**Published:** 2025-05-15

**Authors:** Beate Vajen, Vera Schäffer, Marlies Eilers, Brigitte Schlegelberger, Britta Skawran

**Affiliations:** Department of Human Genetics, Hannover Medical School, Carl-Neuberg-Straße 1, 30625 Hannover, Germany

**Keywords:** *Gonolobus Condurango*, Triple-negative breast cancer, Histone deacetylase inhibitor

## Abstract

**Background:**

Triple-negative breast cancer (TNBC) is a subtype associated with poor prognosis, low survival rates, and high expression of histone deacetylases (HDAC). Treatment with HDAC inhibitors (HDACi) induces the acetylation of histones and thereby the expression of tumor suppressive miRNAs that regulate proliferation, apoptosis, migration, and differentiation. *Gonolobus condurango* (GC) has been reported to exhibit HDAC inhibitory effects, and this study aims to investigate whether GC acts as a HDACi in TNBC cell lines.

**Methods:**

Expression and acetylation analyses were performed on the TNBC cell lines HCC38, HCC1395, and HCC1937. Cells were treated with HDAC inhibitors Trichostatin A (TSA), suberoylanilide hydroxamic acid (SAHA), or Romidepsin as well as with GC Urtincture and different dilutions of GC. Tumor-relevant functional effects were analyzed using WST-1-based proliferation and Caspase-3/7 based apoptosis assays. Induction of expression of tumor-suppressive miRNAs hsa-miRNA-192-5p (miR-192) and hsa-miR-194-2 (miR-194) was analyzed by qRT-PCR.

**Results:**

Meta-analyses of gene expression showed a significant reduction in *HDAC1* and *HDAC2* expression in triple-negative breast cancer samples. The TNBC cell lines (HCC38, HCC1395, and HCC1937) used for in vitro assays also exhibited reduced expression of *HDAC1*,* HDAC2*,* HDAC3*, and *HDAC4* and low acetylation levels. Treatment with the HDAC inhibitors TSA, SAHA, or Romidepsin induced acetylation, while GC did not. TSA and GC Urtincture induced apoptosis in HCC38, whereas GC dilutions had no effect. Treatment with TSA forced the expression of tumor suppressive miRNAs miR-192 and miR-194, but neither GC Urtincture nor any GC dilution induced the expression of these miRNAs.

**Conclusion:**

Several classes of HDAC inhibitors have been shown to be potent and specific anticancer agents. In this study, *Gonolobus condurango* showed no HDAC inhibitory effect in the TNBC cell lines. Identifying new HDAC inhibitors is important, but they must be well characterized before being used as therapeutic agents in humans.

**Supplementary Information:**

The online version contains supplementary material available at 10.1186/s12906-025-04896-w.

## Background

Triple-negative breast cancer (TNBC) is a subtype of breast cancer that is characterized by the absence of estrogen and progesterone receptor expression as well as the absence of overexpression or amplification of HER2, an epidermal growth factor receptor [1]. TNBC is known to be aggressive and is associated with a poor prognosis [2]. Currently, there are limited therapeutic options available for the treatment of TNBC [3]. Therefore, discovering new treatment options to improve the prognosis and outcomes for patients with TNBC is crucial.

Histone deacetylases (HDACs) are overexpressed in many types of tumor, resulting in hypoacetylation in different regions of the genome that leads to reduced transcription and is associated with poor prognosis [4]. Histone deacetylase inhibitors (HDACis) can reverse this effect and were found to decrease cell viability and increase apoptosis of different types of cancer cells but not in normal cells [5]. Despite their benefits, which were proven in clinical trials, synthetic HDACis have undesirable adverse effects [6]. Therefore, the search for other substances such as natural products and their derivatives that act as HDAC inhibitors is worthwhile.

HDACis can broadly be classified into different structural groups, such as hydroxamic acids, cyclic peptides, benzamides, and short-chain fatty acids [2]. The hydroxamates include, for example, SAHA (Vorinostat), Givinostat, Abexinostat, Panobinostat, Belinostat, and the prototypical HDACi Trichostatin A (TSA); cyclic peptides include compounds such as Romidepsin/FK228 and Trapoxin; benzamides include Entinostat and Mocetinostat; the short-chain fatty acids include Valproic acid and Butyrate. SAHA and Romidepsin received approval from the US FDA in 2006 and 2009, respectively. Several ongoing phase I and II studies are investigating HDACi as monotherapy but also in combination with chemotherapeutics for TNBC [7].

Treatment with HDACis leads to altered gene expression, but it may also reduce microRNA (miRNA) expression. These short, non-coding RNAs regulate gene expression post-transcriptionally by binding to complementary sites in 3´ untranslated regions (3’ UTRs) of specific target mRNAs [8]. Global downregulation of miRNAs results in dysregulated proliferation, apoptosis, and differentiation [9]. We previously reported that treatment with HDACis, such as TSA, increased acetylation and induced expression of the tumor-suppressive miR-449 family and miR-192/miR-194 in triple-negative breast cancer [10, 11].

Therapeutics used in traditional and complementary medicine, such as *Gonolobus condurango*, appear to be epigenetically active. *Gonolobus condurango*, also known as *Marsdenia condurango*, belongs to the plant family Asclepiadaceae and is native to tropical regions worldwide [12]. The bark of this plant is used worldwide as a remedy for various diseases, such as stomach ulcers, inflammation of the esophagus, or nutritional disorders [13]. The extract contains a group of novel glycosides and steroids, along with tannin, small quantities of a strychnine-like alkaloid, caoutchouc, condurangin, condruit, essential oil, phytosterin, resin, and sitosterol [14].

The GC extract has a strong toxic effect. The working group of Bishayee et al. demonstrated that the toxic effect of GC increased with its concentration and was due to an increase in reactive oxygen species (ROS) [14, 15]. By adding, for example, N-acetyl cysteine, a scavenger of ROS, the toxic effect of GC was prevented. The working group of Saha et al. showed that an ultra-highly diluted GC extract C30 changed gene expression significantly after administration, by global genetic analyses [16]. *Gonolobus condurango* C30 has also been shown to alter genome methylation [17, 18]. In addition to the effect on methylation, GC C30 affected acetylation and was identified as an HDAC2 inhibitor in the cervical cancer cell line HeLa [19]. Therefore, we investigated the role of GC as an HDAC inhibitor in different dilutions and compared it with known HDACis across different TNBC cell lines.

## Methods

### Cell culture and treatment

To analyze the effect of GC in breast cancer, the following cell lines were used: HCC38, HCC1395, HCC1937, MCF12A, and HeLa. All cell lines were obtained from the American Type Culture Collection in Manassas, USA. HCC38 (CRL-2314), HCC1395 (CRL-2324), and HCC1937 (CRL-2336) are all cell lines of the breast cancer subtype TNBC. We focused on TNBC due to its known aggressiveness and its poor prognosis [2]. For TNBC, there are limited therapeutic options available [3]. The three chosen TNBC cell lines are of hereditary origin and share a family history of cancer. As researchers in human genetics we are mostly interested in hereditary cancers. HCC38 cells are epithelial cells isolated from the mammary gland of a 50-year-old white female with a prior history of leiomyosarcoma; her mother died of breast cancer. HCC38 cells have a pathogenic variant in *TP53* and *CDKN2A*. HCC1395 is an epithelial cell that was isolated from the mammary gland of a 43-year-old, white female patient with ductal carcinoma, TNM Stage I, grade 3. HCC1395 cells have a pathogenic variant in *BRCA1*,* CDKN2A*,* PTEN* and *TP53*. HCC1937 cells were isolated from a primary ductal carcinoma derived from a 23-year-old, white female with stage IIB breast cancer. HCC1937 cells have a pathogenic variant in *BRCA1* and *TP53*. The cells were cultured as recommended by ATCC. Briefly, the triple-negative breast cancer cell lines HCC38, HCC1937, HCC1395 and the cervix carcinoma cell line HeLa (CRM-CCL-2) were cultivated in T25 (Sarstedt #83.3910.002), T75 (Sarstedt #83.3911.002) or T175 (Sarstedt #83.3912.002) cell culture flasks at 37 °C and 5% CO_2_ in a humidified incubator. HCC38 were supplied with RPMI-1640 growth medium (Sigma-Aldrich #R8758) with 10% fetal bovine serum (Merck #S0615), 1% penicillin/streptomycin (Merck #P0781), 12,5 mL 10% D-glucose solution (Sigma-Aldrich #G8644), 1% HEPES (Merck #H0887) and 1% sodium pyruvate (Merck #S8636). HeLa cells were supplied with DMEM (Sigma #D5796), 10% fetal bovine serum (Merck #S0615) and 1% sodium pyruvate (Merck #S8636) and 1% penicillin/streptomycin (Merck #P0781).

All cell lines were tested for mycoplasma contamination by PCR Mycoplasma Test Kit (Promo Cell, Heidelberg, Germany #PK-CA91-1048). For treatment with TSA (330 nM), SAHA (2 µM) or Romidepsin (35 nM) or ethanol vehicle as a control, the medium was renewed every 12 h. Cells were treated with 2% *Gonolobus condurango* mother tincture/ urtincture (Deutsche Homöopathie-Union, Karlsruhe, Germany, Ch.-B.: 1090819) or ethanol vehicle control. To analyze the effect of *Gonolobus condurango* in homoepathic dilutions, dilutions in C6 (Ch.-B.: 330235469), C30 (CH.-B.: 330235547) and D6 (Ch.-B.: 0050720) were bought from DHU (Deutsche Homöopathie-Union, Karlsruhe, Germany). DHU promises products of consistently outstanding quality by following the guidelines of the German Homeopathic Pharmacopoeia (Homöopathisches Arzneimittelbuch, HAB), the regulations of the European Pharmacopoeia (Ph. Eur.) and the guidelines of the European directive on Good Manufacturing Practice (GMP). DHU explains on its website: All liquid medicinal forms, known as dilutions, are succussed– each active ingredient is mixed with an ethanol/water solution at a ratio of 1:10. Potentization is performed exclusively by hand at DHU. Depending on the number of steps, the desired potency is achieved. The following dilutions of *Gonolobus condurango* were bought for this study: D-potencies at a ratio of 1:10 and C-potencies at a ratio of 1:100. The treatment times are based on times already described in the literature.

### Acetylation assay

Global histone acetylation was determined by the Cyclex Cellular Histone Acetylation Kit (#CY-1140 Cyclex Co, Nagano, Japan) according to the manufacturer’s instructions. Briefly, after 24 h treatment of the cancer cell lines with the HDAC inhibitors or GC, the levels of histone acetylation were analyzed. During the assay, the cells are fixed to the sides of the well and permeabilized, followed by a typical treatment of the wells with a blocking reagent and two types of antibodies. The primary antibody (anti-acetylated histone/p53-K382 monoclonal antibody; supplied with the kit) binds to acetylated histones, including H3 and H4. The secondary antibody is specific for the first and has a horse-radish peroxidase conjugated to it which converts the chromogenic substrate tetramethylbenzidine until a stop solution is added. The absorbance is measured at 450 nm wavelength.

### Proliferation and apoptosis assay

Cell viability and apoptosis were measured in triplicate using the WST-1 Proliferation Reagent (#5015944001 Roche, Basel, Switzerland) and the Caspase3/7 Glo Assay (#G8093 Promega, Madison, WI, USA), respectively. The proliferation was analyzed by adding water-soluble tetrazolium-1 (WST-1) which is converted to formazan by metabolic active cells. Formazan was measured at a certain wavelength and the intensity of the signal is proportional to the amount of living cells and therefore serves as measure for cell viability. For the assay, 10 µl WST-1 was added to the 100 µl medium per well and incubated for one hour in the 37 °C CO2 incubator. Using the multi-detection reader Synergy2 (BioTek), the background absorption was measured at 650 nm wavelength and subtracted from the actual absorption values at 450 nm wavelength. These values were normalized to the ethanol control. Apoptosis was analyzed by adding a luminogenic reagent to the growth medium that contained the cancer cells. The effector caspases − 3 and − 7 in the cells split this reagent and the emerging luminescence is proportional to the activity of the two caspases and therefore serves as measure of apoptosis. Caspase3/7 assay was performed on a second 96-wells microtiter-plate by adding 100 µl Caspase-Glo3/7 reagent to 100 µl growth medium. The plate was then incubated at room temperature in the dark for one hour before measuring the luminescence in a white micro-titer plate using the Synergy2 multi-detection reader. The results were normalized to the ethanol control and then also on the values of the WST-1 assay, in order to equalize eventual differences in cell numbers that might have developed during the experiments.

### Analysis of MiRNA expression

To isolate RNA, cells were lysed in Qiazol Lysis Reagent (#79306 Qiagen) and total RNA including miRNAs was isolated with the miRNeasy Mini Kit (#217004 Qiagen). RNA quality was routinely determined using the Bioanalyzer 2100 (Agilent, Santa Clara, CA, USA). Expression of mRNA and miRNA was measured in triplicate by quantitative real-time PCR (qRT-PCR). Complementary DNA (cDNA) was reverse transcribed with the High Capacity cDNA Transcription Kit (#4374967 Life Technologies). For mRNA, cDNA synthesis was performed with 250 ng total RNA using random hexamer primers. For miRNA, Taqman MicroRNA Assays (Life Technologies) were used for synthesis of miRNA-specific cDNA from 100 ng total RNA and qRT-PCR was performed with *RNU6B* as reference gene.

Expression of miRNA was measured in triplicate. The following Taqman Assays were used: hsa-miR-192-5p, AssayID 000491 and hsa-miR-194-5p, AssayID 000493 (Thermo Fisher Scientific, Langenselbold, Germany).

### Statistics

**Fig. 1:**
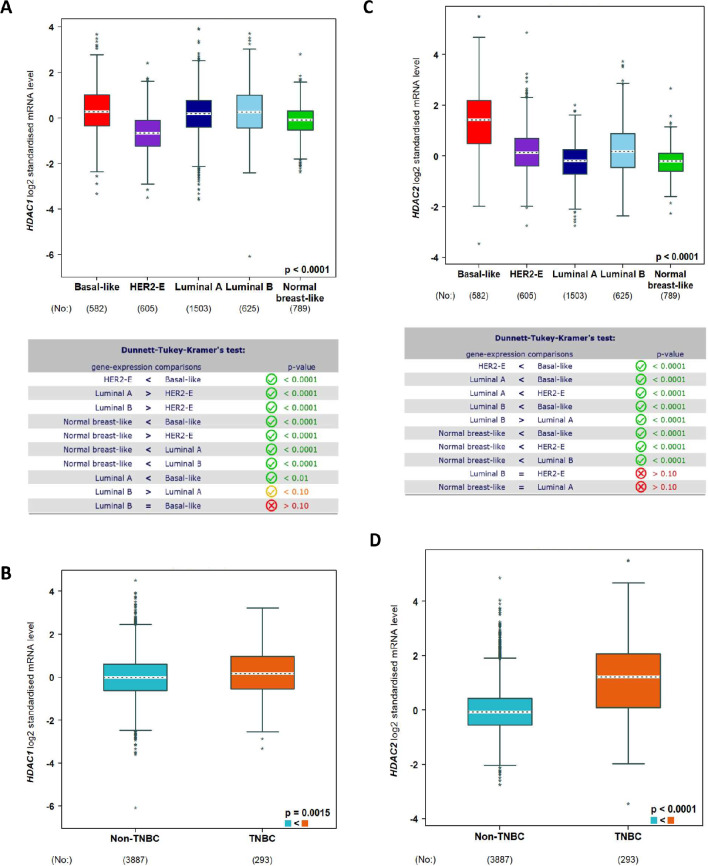
Expression of HDAC1 and HDAC2 in public datasets. Expression levels of HDAC1 (**A**, **B**) and HDAC2 (**C**, **D**) analyzed using the public data sets SCAN-B GSE96058, SCAN-B GSE81538 and TCGA with *n* = 4421. Box and whisker plots (**A**, **C**) show expression according to Sorlie´s subtypes and the results of the Dunnett-Tukey-Kramer test to analyze the significant differences in expression between the groups (box below). Bow and whisker plots (**B**, **D**) show expression according to TNBC status. The results of HDAC3 and HDAC8 are presented in supplementary Figure S1. Figures were created by using bc-GenExMiner 4.8 [21]. Statistical test: 1-way ANOVA, Dunnett-Tukey-Kramer posthoc-test or Student´s t-test

Data are represented as mean ± SD of at least three independent experiments. Statistical significance was determined by 2-tailed Student’s *t* test, by 1-way ANOVA followed by Dunnett’s multiple comparison test or by 2-way ANOVA with the Geisser-Greenhouse correction. All statistical analyses were completed with the GraphPad Prism software 8 (GraphPad Software, La Jolla, CA, USA). Figure 1 and Figure S1 were created by bc-GenExMiner 4.8 [20], a statistical mining tool of published annotated breast cancer transcriptomic data from DNA microarrays and RNA-sequencing.

## Results

### Expression of histone deacetylase HDAC1 and HDAC2 is significantly reduced in triple-negative breast cancer

HDACs are found to be overexpressed in breast cancer and various other tumors, which reduce the general level of acetylation in breast cancer cells [4]. The inhibition of HDACs from class I (HDAC 1, 2, 3 and 8) showed severe anti-proliferative and proapoptotic effects [21]. To analyze the expression of class I HDACs in different breast cancer subtypes and normal breast-like tissue, we performed meta-analyses on gene expression using bc-GenExMiner 4.8 [20], a statistical mining tool of published annotated breast cancer transcriptomic data from DNA microarrays and RNA-sequencing. For the expression analyses of HDAC1, HDAC2, HDAC3 and HDAC8 the following datasets SCAN-B GSE96058 [22], SCAN-B GSE81538 [23] and TCGA [24] with *n* = 4421 were used. The different breast cancer subtypes defined by different expression patterns by Sorlie et al. [25] showed a significantly different expression of HDAC1 compared to normal breast-like tissue (Fig. 1A).

The expression of HDAC1 was significantly increased in the subtypes Basal-like, Luminal A and Luminal B compared to normal breast like samples. Comparing non-TNBC and TNBC samples, we observed a significantly increased expression of HDAC1 in TNBC samples (Fig. 1B). HDAC2 is highly expressed in Basal-like samples compared to normal breast like samples (Fig. 1C). Furthermore, HDAC2 expression is significantly increased in HER2-E and Luminal B subtypes compared to the expression in normal breast like samples. In TNBC samples the expression of HDA2 is significantly higher compared to non-TNBC samples (Fig. 1D). HDAC3 expression differs between the subtypes (Fig. S1A). In the subtype Luminal A, we observed a significant higher expression of HDAC3, but not in HER2-E, Luminal B and Basal-like. The expression of HDAC3 is decreased in TNBC compared to the expression in non-TNBC samples (Fig. S1B). The expression of HDAC8 is significantly higher in Luminal A and Basal-like subtype, but is decreased in HER2-E and the same in Luminal B compared to the expression in normal breast like samples (Fig. S1C). There was no different expression of HDAC8 between TNBC and non-TNBC samples (Fig. S1D).

### HDAC1, HDAC2, HDAC3 and HDAC4 expression is higher and the acetylation level is lower in TNBC cell lines compared to the breast cell line MCF12A

**Fig. 2:**
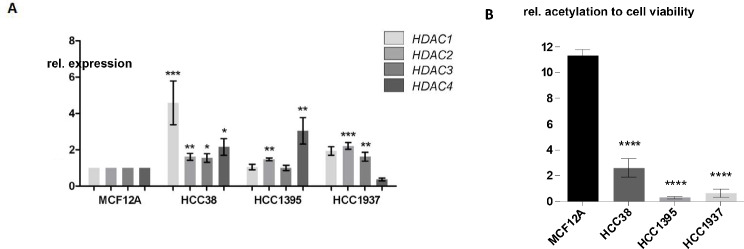
High expression of HDAC1 to HDAC4 and low acetylation level in TNBC cell lines. **A**) HDAC1, HDAC2, HDAC3 and HDAC4 expression on mRNA level in TNBC cell lines HCC38, HCC1395 and HCC1937 compared to the non-tumorigenic breast cell line MCF12A. **B**) Histone acetylation of HCC38, HCC1937 and HCC1395 compared to the non-tumorigenic breast cell line MCF12A. The results were normalized to cell viability measured by WST-1 assay. *P < 0.05, **P < 0.01, ***P < 0.001, ****P < 0.0001, 1-way ANOVA / Dunnett’s multiple comparison test

Since HDACs are often overexpressed in samples of TNBC patients, we wanted to investigate if this is also true for the TNBC cell lines HCC38, HCC1395 and HCC1937. We observed significantly induced expression of HDAC1, HDAC2, HDAC3 and HDAC4 in HCC38 cells compared to the spontaneously immortalized breast cell line MCF12A that was derived from breast epithelium (Fig. 2A).

HCC1395 showed significantly higher expression of HDAC2 and HDAC4 compared to the expression measured in MCF12A. Furthermore, the expression of HDAC2 and HDAC3 are significantly induced in HCC1937 compared to the expression in MCF12A cells.

Since an overexpression of HDACs leads to reduced acetylation, we were interested in the acetylation status of the TNBC cell lines HCC38, HCC1395, HCC1937 and the breast cell line MCF12A. For this purpose, we performed acetylation assays. The TNBC cell lines showed significantly reduced acetylation levels compared to the immortalized breast cell line MCF12A (Fig. 2B).

### Histone deacetylase inhibitor TSA increased in contrast to GC the acetylation of histones in HCC38 cells

It is well known that HDACi such as TSA, SAHA or Romidepsin reduce HDAC activity significantly. TSA and SAHA inhibit the activity of HDACs of class I, class II and class IV [26]. Romidepsin is a more specific inhibitor and inhibits class I HDACs [27]. The inhibition of HDACs leads to a higher level of acetylation and can even lead to apoptosis of cancer cells. We proofed this for the TNBC cell line HCC38 and observed an increased acetylation (Fig. S2A), a reduced proliferation (Fig. S2B) and increased apoptosis after treatment with TSA, SAHA as well as Romidepsin (Fig. S2C).

**Fig. 3:**
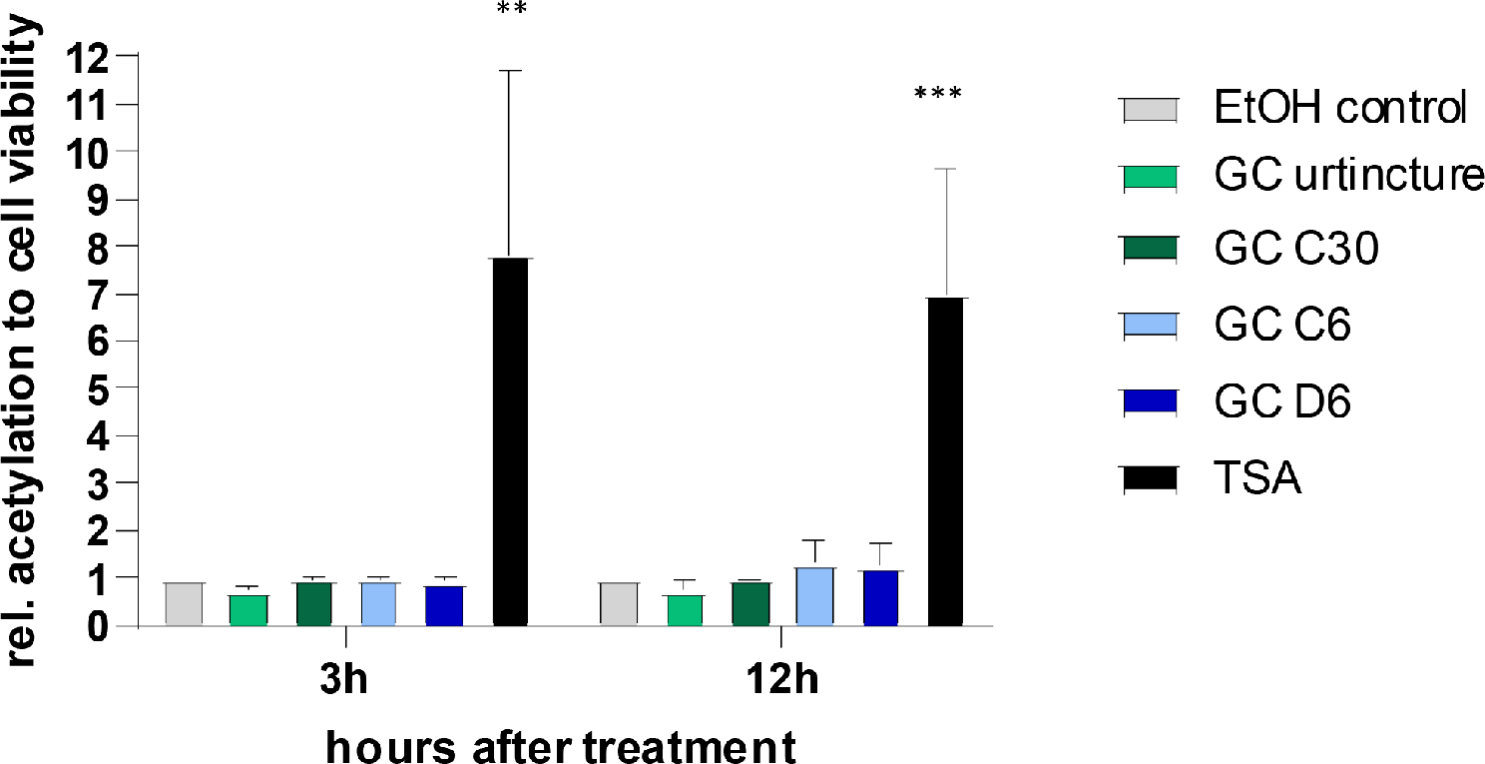
Acetylation levels of HCC38 cells treated with GC Urtincture and different dilutions of GC. Histone acetylation of HCC38 after treatment with GC Urtincture, GC C30, GC C6, GC D6 or with TSA as a positive control. The results were normalized to the ethanol controls and the viability of cells measured by WST-1 assay. **P < 0.01, ***P < 0.001, 2-way ANOVA

*Gonolobus condurango* has been described to induce acetylation as well [18, 19]. To analyze the role of GC as an HDACi, we first performed an acetylation assay with the Urtincture as well as the dilutions GC C6, C30 and D6 and used TSA as a positive control. TSA already increased the acetylation of histones in HCC38 cells significantly after three hours of treatment (Fig. 3).

The GC Urtincture as well as the dilutions GC C6, GC C30 and GC D6 did not increase the acetylation after either three or twelve hours in HCC38 cells. Since it has been published that GC inhibits HDAC2 in HeLa cells [19], we additionally analyzed the acetylation in HeLa cells (Fig. S3). In contrast to TSA, no induction in acetylation by GC in HeLa cells was detectable.

### In contrast to dilutions of GC, TSA and GC urtincture induced apoptosis in HCC38

**Fig. 4:**
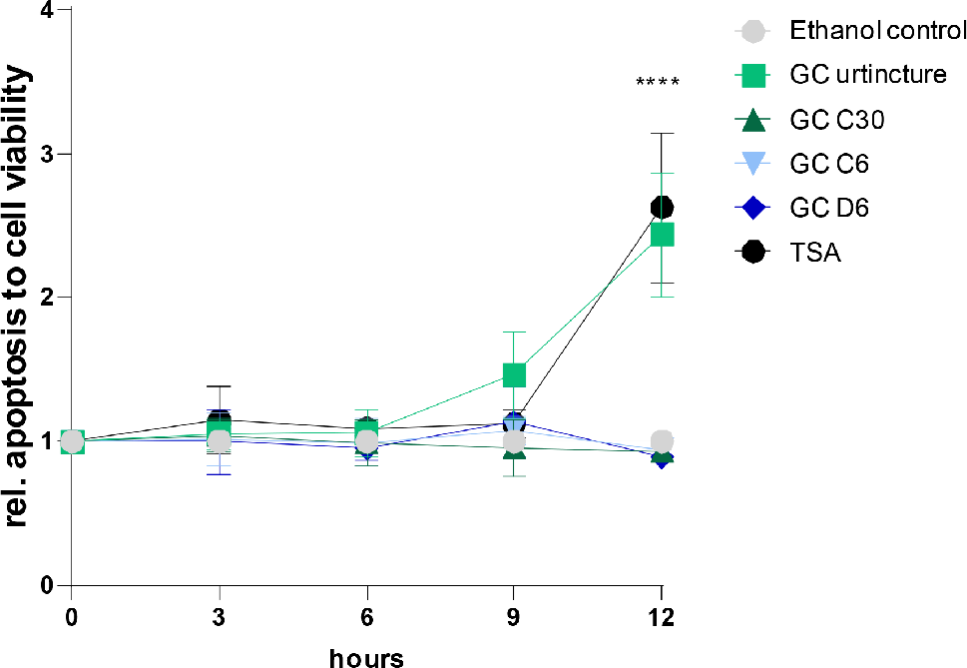
Apoptosis of HCC38 cells treated with GC Urtincture and different dilutions of GC. Apoptosis analyzed by caspase3/7 activity was normalized to cell viability. HCC38 cells were treated with GC Urtincture, GC C30, GC C6, GC D6 or with TSA (as a positive control) and normalized to the ethanol control (grey line). ****P < 0.0001, 2-way ANOVA

In order to investigate tumor suppressive effects of GC in vitro, we performed proliferation and apoptosis assays. After twelve hours, we observed a significant increase in apoptosis for HCC38 cells treated with TSA and the GC Urtincture (Fig. 4).

The relative apoptosis was 2.5-fold induced in HCC38 cells after treatment with TSA or GC Urtincture. The dilutions GC C6, GC D6 or GC C30 did not increase apoptosis in HCC38 within twelve hours of treatment. Furthermore, there was no induction in apoptosis in HeLa cells detectable (Fig. S4).

### In contrast to GC, TSA induced the expression of tumor suppressive miR-192 and miR-194

**Fig. 5:**
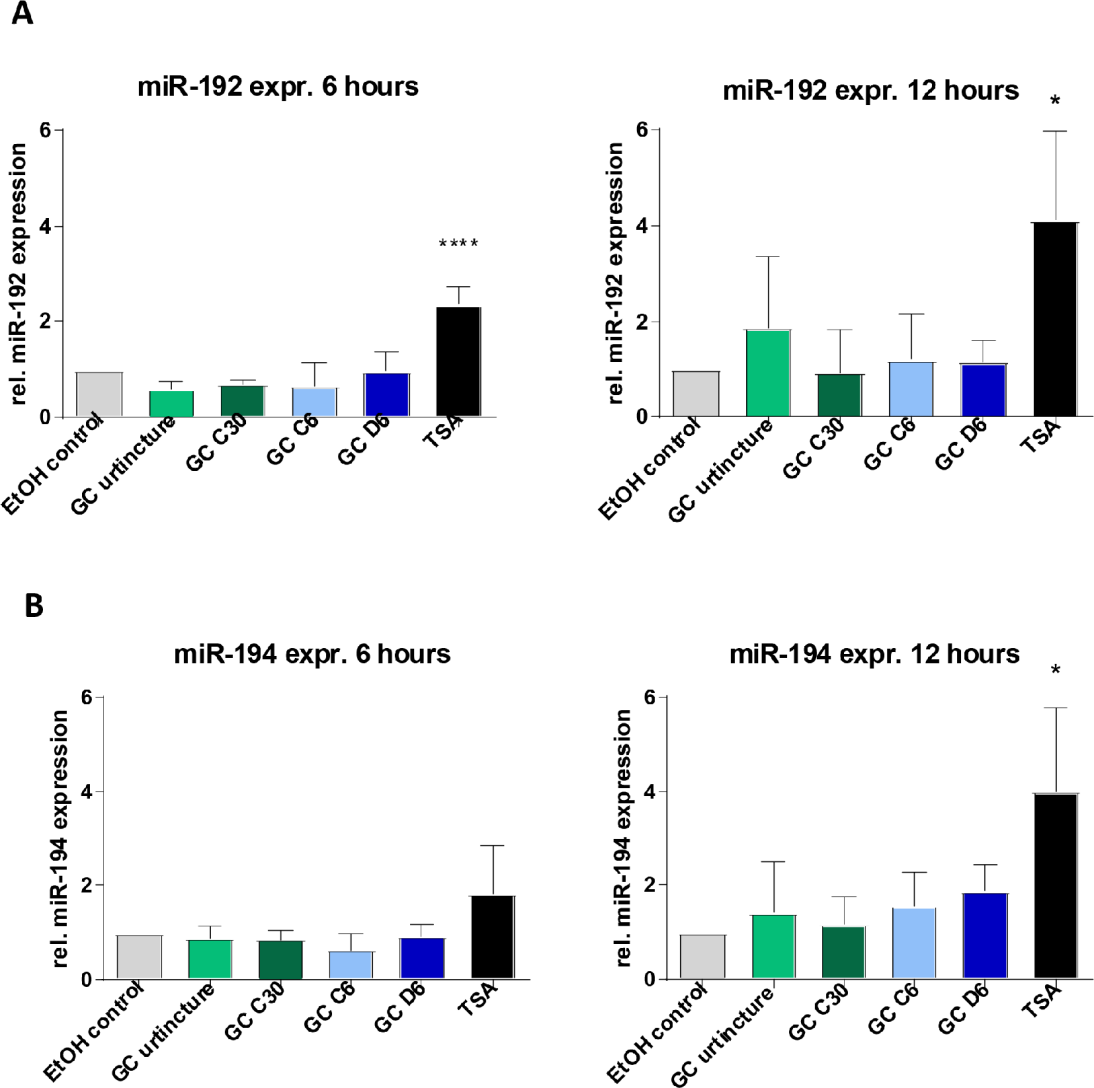
Expression of tumor suppressive miR-192 and miR-194 of HCC38 cells treated with GC Urtincture and different dilutions of GC. **A**) miR-192 expression on mRNA level six hours (left) and twelve hours (right) of HCC38 cells normalized to ethanol control. **B**) miR-194 expression on mRNA level six hours (left) and twelve hours (right) of HCC38 cells normalized to ethanol control. The results of additional time points are presented in Supplementary Fig. S4. * *P* ≤ 0,05; **** *P* ≤ 0,0001, 1-way ANOVA / Dunnett’s multiple comparison test

The inhibition of HDACs leads to an increased level of acetylation followed by an increased transcription of genes including microRNAs. The re-expression of tumor suppressive microRNAs is jointly responsible for the anti-proliferative effects of HDAC inhibitors. Therefore, we analyzed the expression of two epigenetically regulated tumor suppressive miRNAs, miRNA-192 and miRNA-194, after treatment with GC, GC dilutions and TSA. Treatment with the positive control TSA induced the expression of miR-192 after six hours as well after 12 h (Fig. 5A).

Furthermore, we observed a significant increase in miR-194 expression after six hours as well as after twelve hours of TSA treatment (Fig. 5B). GC Urtincture or the dilutions, however, had no effect on the expression of tumor suppressive miRNA-192 (Fig. S5A) or miR-194 (Fig. S5B) in HCC38 cells. Furthermore, there was no induction of miRNA expression in HeLa cells detectable by GC (Fig. S5C, S5D).

## Discussion

Cancer cells are characterized by their ability to survive even under difficult conditions, making them highly resilient. Their increased cell viability is attributed to reduced histone acetylation, leading to a decrease in transcriptional activity [28]. Studies have shown that HDACis such as TSA, SAHA, and Romidepsin can reduce cell viability and induce apoptosis in various types of cancer cells without affecting normal cells [29]. Given the success of HDAC inhibition as treatment strategy, it is worthwhile to investigate other substances that could function as HDAC inhibitors.

To investigate whether altered HDAC expression is also involved in TNBC, we first analyzed the expression of *HDAC1*,* HDAC2*,* HDAC3*, and *HDAC8* in publicly available datasets. We observed increased expression of *HDAC1* and *HDAC2* in TNBC samples compared to non-TNBC samples. *HDAC3* expression was lower in TNBC samples than in non-TNBC samples. *HDAC8* showed no significant differences in expression between TNBC and non-TNBC samples. These findings are consistent with those of Müller et al. [30], who showed that the expression of class 1 HDAC isoenzymes is deregulated in breast cancer samples, and that varying expression levels of *HDAC1*, *HDAC2* and *HDAC3* correlated with clinicopathological parameters.

Moreover, our analysis revealed an increased expression of HDAC1, HDAC2, HDAC3, and HDAC4 in the TNBC cells used in this study, as compared to the immortalized breast cell line MCF12A. This increased expression was correlated with a reduction in acetylation levels in the TNBC cells. This finding made our cell lines a suitable model for investigating the potential impact of HDACis on TNBC.

A recent review analyzing the role of natural products in drug development over the past 40 years highlights that natural products and their synthetic derivatives remain a crucial source of new drugs, particularly in the field of cancer [31]. Plants can be used therapeutically in a variety of forms, such as tea, extracts, and dyes. In addition, their active compounds may be isolated and used as medicines or as precursors for synthetic and semi-synthetic drugs [32].

*Gonolobus condurango*, particularly its bark, is widely used as a natural remedy for various diseases [13]. Recent studies indicate that ethanolic extracts of GC possess cytotoxic properties and demonstrate potential anticancer activity [33]. Furthermore, GC war described to inhibit HDAC2 activity [19].

Treatment with HDACi is known to result in hypoacetylation in various regions of the genome and the repression of transcription [28]. In the TNBC cell lines that we used in this study, we validated the effects of HDACi treatment and measured an increase in acetylation by the well-known HDACi TSA, SAHA, and Romidepsin. Although GC was described to inhibit the HDAC2 [19], we did not observe an induction in acetylation by treatment with GC Urtincture or GC dilutions, neither in HCC38 nor in HeLa cells. This could be explained by the fact that the inhibition of a single enzyme did not reflect in the overall acetylation measurement.

By analyzing proliferation and apoptosis, we demonstrated the tumor-suppressive role of TSA in the HCC38 TNBC cell line. Additionally, we validated the toxic effect of GC urtincture, which has been previously described by Bishayee et al. [14, 15]. We observed a significant increase in apoptosis twelve hours after treatment with both TSA and GC urtincture. However, we did not observe any significant difference when cells were treated with different GC dilutions compared to the ethanol-treated control. This contrasts with the observation of Bishayee et al. [19], who has found a significant increase in apoptosis in HeLa cells treated with GC C30 for twelve hours, as measured by MTT assay. Although this is a different method for analyzing apoptosis, similar results would be expected. Therefore, it would be advisable to confirm the results in further independent studies.

Furthermore, we observed an increased expression of the tumor suppressive miRNAs, miR-192 und miR-194, in the HCC38 cell line after treatment with TSA, but not with GC. In our previous publications, we have demonstrated that treatment with HDACis, such as TSA, SAHA, and Romidepsin, resulted in an increased expression of tumor suppressive miRNAs due to an increased acetylation already after six hours [10, 11]. Since we did not observe an increase in acetylation by GC treatment, it was expected that GC would not induce the expression of epigenetically regulated tumor suppressive miRNAs. In summary, we could not validate the proposed role of *Gonolobus condurango* as an HDACi using our established methods and compared to well-described HDACis, such as TSA in this study.

## Limitations

Although this study provides valuable insights into the role of HDACi in TNBC cell lines, the shortcomings and limitations of the study need to be considered.

In vitro assays have several limitations, since they are conducted in a controlled environment without the complex interactions that occur in a living organism. They don’t account for systemic factors like the immune system, metabolism, and interactions with other organs that can influence the outcome of a drug. In this study, we used cell lines that may not perfectly represent the heterogeneity and complexity of human tissue. The conditions we conducted, including culture media, temperature, and environment, might not exactly mirror the physiological conditions, leading to potential discrepancies in how a substance would behave under natural biological conditions. This means that the results may not fully replicate how the substance behaves within a whole organism. Furthermore, in vitro studies do not provide information on how a compound is absorbed, distributed, metabolized, or excreted in the body, which is crucial for understanding its potential efficacy and safety in vivo.

Since studies on *Gonolobus condurango* as a potential HDAC inhibitor used the urtincture or homeopathic dilutions, we performed our assays using the same preparations. However, it is also conceivable to perform the experiments with the isolated active constituent of GC. The active constituents of GC extracts were described by Bishayee et al. [14] as glycoside-based condurangogenins, also known as condurango glycosides.

To compare our results with those from literature, we performed proliferation and survival assays after 3, 6, 9 and 12 h as described by Bishayee et al. [19]. To analyze long-term effects of GC treatment on proliferation and survival, assays could be performed after 24–48 h. For the same reason, we decided to use C30 of GC. To further characterize additional homeopathic dilutions, we selected another high potency, C6, and a potency D6, in which traces of the original substance are still present. Since we cannot exclude a dose-dependent effect, further experiments with different concentrations could be conducted.

To further explore the HDAC inhibitory effect of GC in a broader range of TNBC models, a wider panel of TNBC cell lines representing different molecular subtypes (e.g., basal-like, mesenchymal, or luminal morphology) could be studied. To analyze epigenetic changes, additional Western blot analyses (e.g., H3K9ac, H4K16ac) could have been conducted as previously described [10].

In previous studies, we performed RNA sequencing experiments to identify gene expression changes [11]. Since we detected no effect of GC in the experiments presented in this study, we did not conduct gene expression profiling. To analyze anti-tumor effects, proliferation and apoptosis were measured in this study. In previous studies, we also examined the migration behavior of the cells by transwell migration assays [11].

Moreover, the in vivo behavior of treated cancer cells could be analyzed in xenograft models. Although we successfully established a xenograft model for HDACi-treated hepatocellular carcinoma cell lines [34], we have not established a mouse model for the TNBC cell lines used in this study. Other TNBC cell lines, such as MDA-MB-231, are better suited for this purpose [35].

Currently, several HDAC inhibitors have been approved by FDA for the treatment of hematological malignancies, including vorinostat (SAHA), belinostat (PXD-101), romidepsin (FK-228) and Panobinostat (LBH589) [36]. However, HDAC inhibitors have shown limited success in the treatment of solid tumors [37]. In contrast, HDACi treatment combined with other promising agents could show a synergistic effect. The combined use of PARPis and HDACis has been shown to mediate immunomodulatory functions and represents a potential therapeutic approach for TNBC [38]. In a previous publication, we also demonstrated that combining a tumor-suppressive miRNA—epigenetically upregulated by HDACi treatment—with a PARPi enhanced the anti-cancer effect [10]. Since we did not observe any differences in acetylation or miRNA expression, no further experiments were conducted to combine different agents.

## Conclusions

The inhibition of HDACs has emerged as a promising strategy in recent years for reversing aberrant epigenetic changes associated with cancer. Several studies have found that different classes of HDAC inhibitors exhibit potent and specific anticancer activities. In this study, *Gonolobus condurango* showed no HDAC inhibitory effect. While it is worth identifying new HDACi, it is important to thoroughly characterize them before use. The findings of this study suggest that future research on HDACis should focus on identifying and testing novel HDACis, particularly in combination with other therapies, such as PARPis, to enhance treatment efficacy in TNBC and potentially overcome the limitations observed in solid tumor therapies.

## Electronic supplementary material

Below is the link to the electronic supplementary material.

Supplementary Material 1

## Data Availability

The datasets used and analysed during the current study are available from the corresponding author on request.

## Abbreviations

ATCC: American Type Culture Collection
DNA: Deoxyribonucleic acid
GC: Gonolobus condurango
HDAC: Histone deacetylase
HDACi: Histone deacetylase inhibitor
miR-192 has: microRNA-192-5p (miR-192)
miR-194 ahsa: microRNA-194-2
PBS: Phosphate Buffered Saline
qRT: PCR Quantitative Real Time PCR
RNA: Ribonucleic acid
SAHA: Suberoylanilide hydroxamic acid
SD: Standard deviation
TNBC: Triple-negative breast cancer
TSA: Trichostatin A
3’UTR 3’: Untranslated region
WST: Water soluble tetrazolium
