# Supplementary material for: Exploring the potential of Gonolobus condurango as a histone deacetylase inhibitor in triple-negative breast cancer cell lines: in vitro study
Source: BMC Complement Med Ther. 2025 May 15;25:177. doi: 10.1186/s12906-025-04896-w (PMC12079997; doi:10.1186/s12906-025-04896-w)
Supplement: Supplementary file 1 — Supplementary Material 1 [file 12906_2025_4896_MOESM1_ESM.docx]

**A**

**Figure S1**


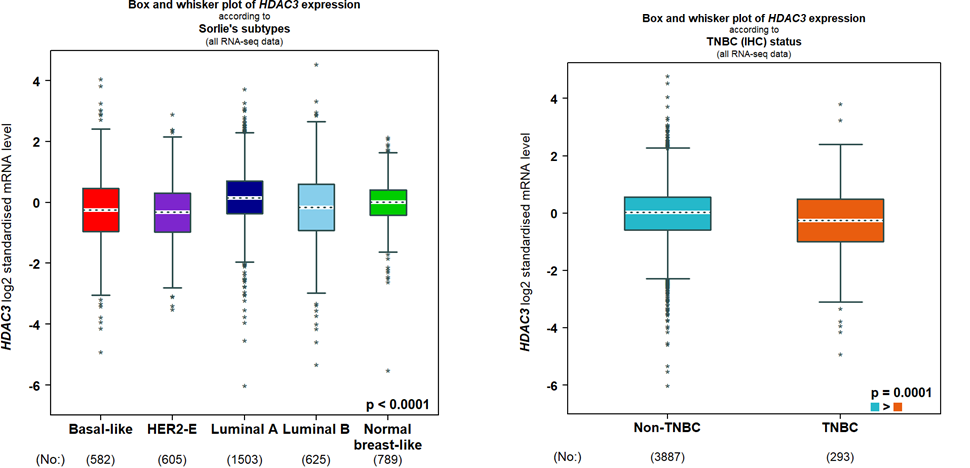


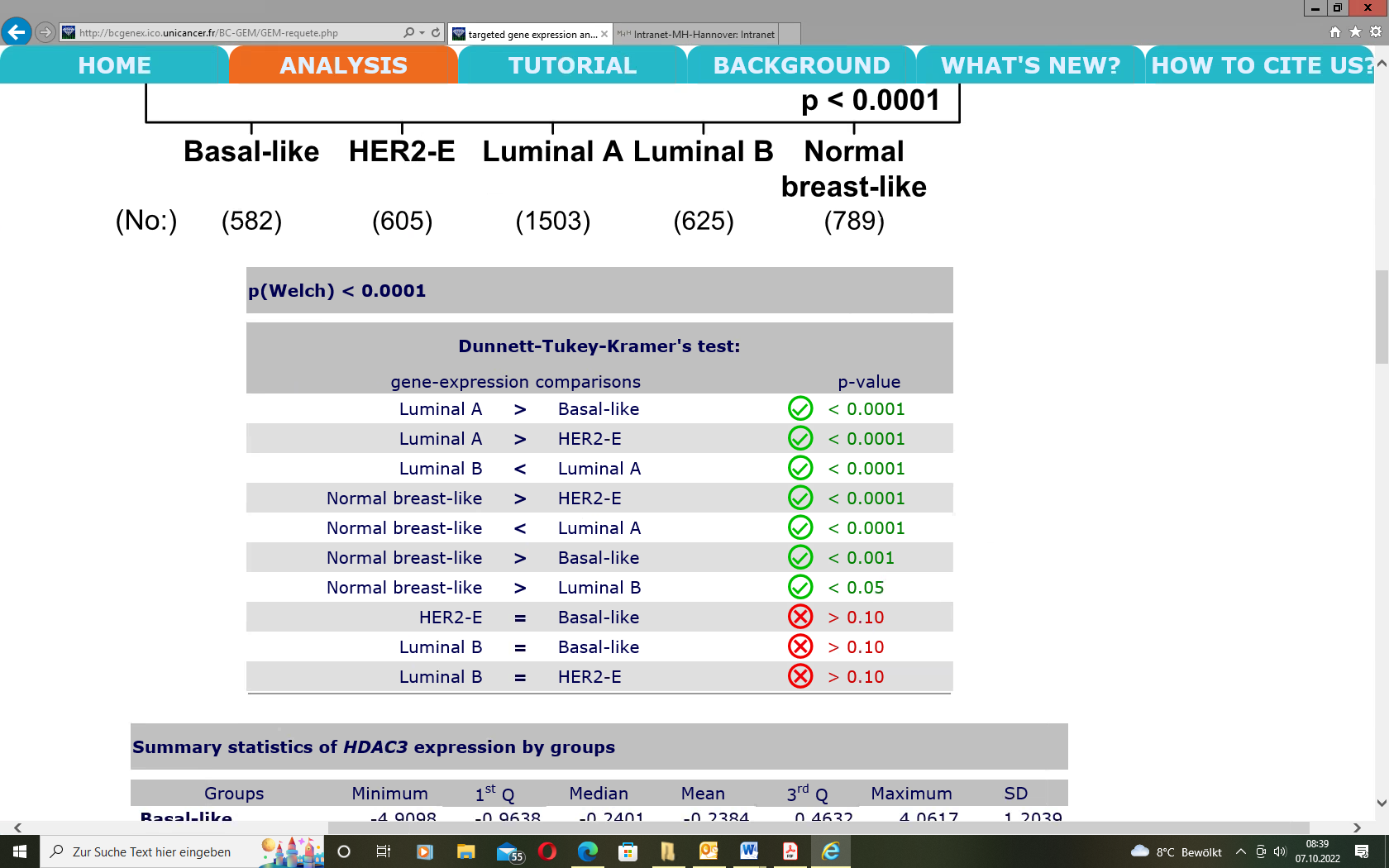


**B**


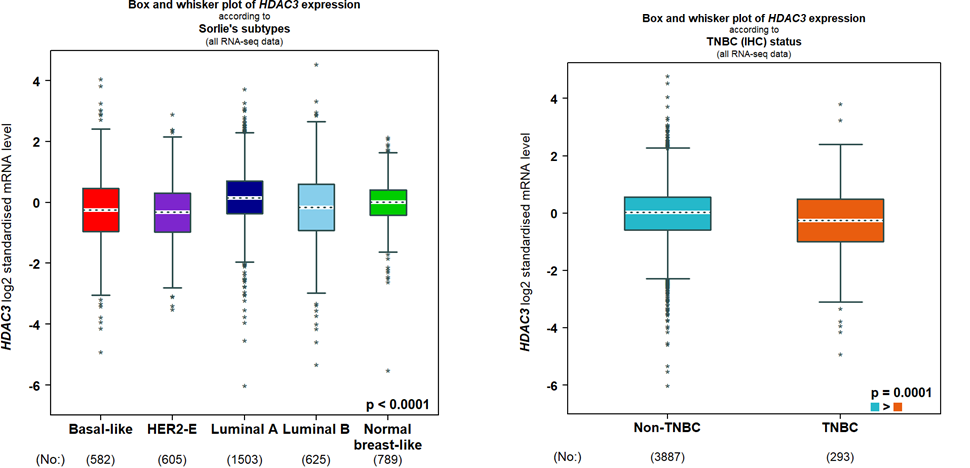


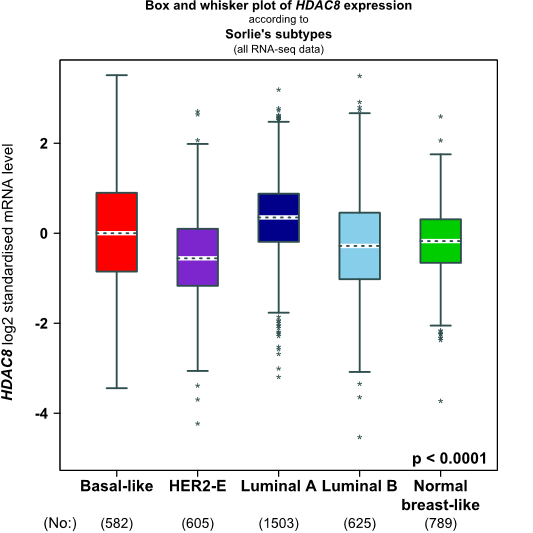


**C**


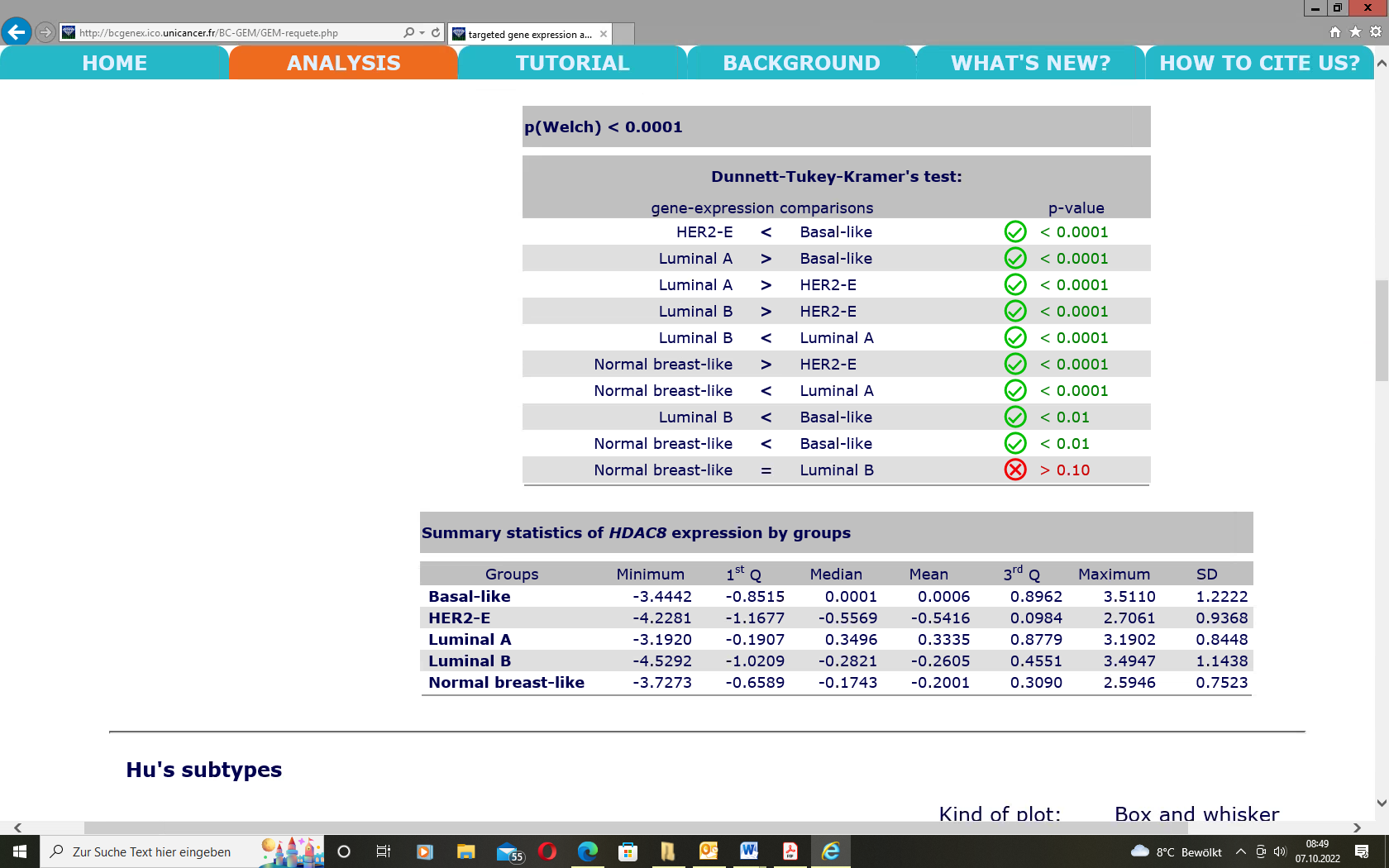


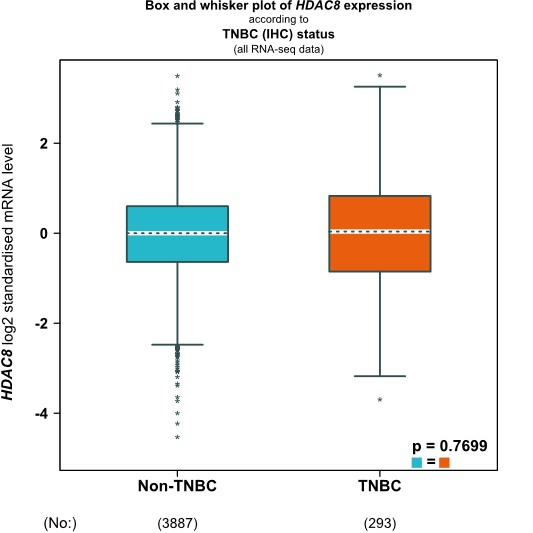


**D**

**Fig. S1: Expression of HDAC3 and HDAC8 in public datasets**

Expression levels of HDAC3 (A, B) and HDAC8 (C, D) analyzed using the public data sets SCAN-B GSE96058^21^, SCAN-B GSE81538 ^22^ and TCGA^23^ with n=4421. Box and whisker plots (A, C) show expression according to Sorlie´s subtypes^24^ with results of Dunnett-Tukey-Kramer posthoc-test to analyze significant differences (box below). Box and whisker plots (B, D) show expression according to TNBC status. Statistical test: 1-way ANOVA, Dunnett-Tukey-Kramer posthoc-test or Student´s t-test.

**2A**

**Figure S2**

**Romidepsin**

**B**

**Romidepsin**

**C**

**Romidepsin**

**Fig. S2: HDACis TSA, SAHA and Romidepsin increased acetylation, decreased proliferation and induced apoptosis of HCC38 cells**

HCC38 cells were treated with TSA, SAHA or Romidepsin and normalized to the ethanol control. Results of acetylation assay (A) as well as of apoptosis assay (C), analyzed by caspase3/7 activity, were normalized to cell viability (WST assay (B)). **P* < 0.05, ***P* < 0.01, ****P* < 0.001, 1-way ANOVA.

**Figure S3**

**

**

**Fig. S3: TSA induces acetylation in HeLa cells.**

The results were normalized to cell viability measured by WST-1 assay. **P < 0.01, 1-way ANOVA / Dunnett’s multiple comparison test.

**Figure S4**

**Fig. S4: Treatment of GC urtincture or different dilutions of GC showed no effect on apoptosis in HeLa cells**

HeLa cells were treated with GC urtincture or GC dilutions GC C30, GC C6, GC D6 or with TSA as a positive control. Results were normalized to the ethanol control. 2-way ANOVA.

**A**

**Figure S5**

**B**

**C**

**D**

**Fig S5: Expression of tumor suppressive miR-192 and miR-194 of HCC38 and HeLa cells treated with GC urtincture and different dilutions of GC**

A) miR-192 and miR-194 (B) expression on mRNA level three hours (left) and nine hours (right) of HCC38 cells treated with GC urtincture or GC dilutions normalized to ethanol control. C) miR-192 and miR-194 (D) expression on mRNA level three hours (left) and nine hours (right) of HeLA cells normalized to ethanol control.

* P ≤ 0,05; ** P ≤ 0,01, 1-way ANOVA / Dunnett’s multiple comparison test.
